# P-1872. Dalbavancin workflow for inpatient discharges: a multidisciplinary model

**DOI:** 10.1093/ofid/ofae631.2033

**Published:** 2025-01-29

**Authors:** Maria Baldino, Alexandra Hanretty, Geena Kludjian, Lisa Pedroza, Raquel Nahra

**Affiliations:** Cooper University Hospital, Camden, New Jersey; Cooper University Hospital and Cooper Medical School of Rowan University, Philadelphia, Pennsylvania; Cooper University Hospital and Cooper Medical School of Rowan University, Philadelphia, Pennsylvania; Cooper Medical School Rowan University, Camden, New Jersey; Cooper University Health, Camden, New Jersey

## Abstract

**Background:**

The role of dalbavancin (DAL) for the treatment of deep-seated infections is of growing interest. The long half-life, extensive tissue distribution, and ease of administration makes it ideal for use in populations that have physical or social barriers to treatment with standard antibiotics for infections such as endocarditis, septic arthritis, and osteomyelitis. A workflow for DAL in high-risk populations has previously not been well described. Our aim is to describe the multidisciplinary approach to DAL on discharge and describe impact on readmissions, length of stay and adherence.

**Figure 1:**
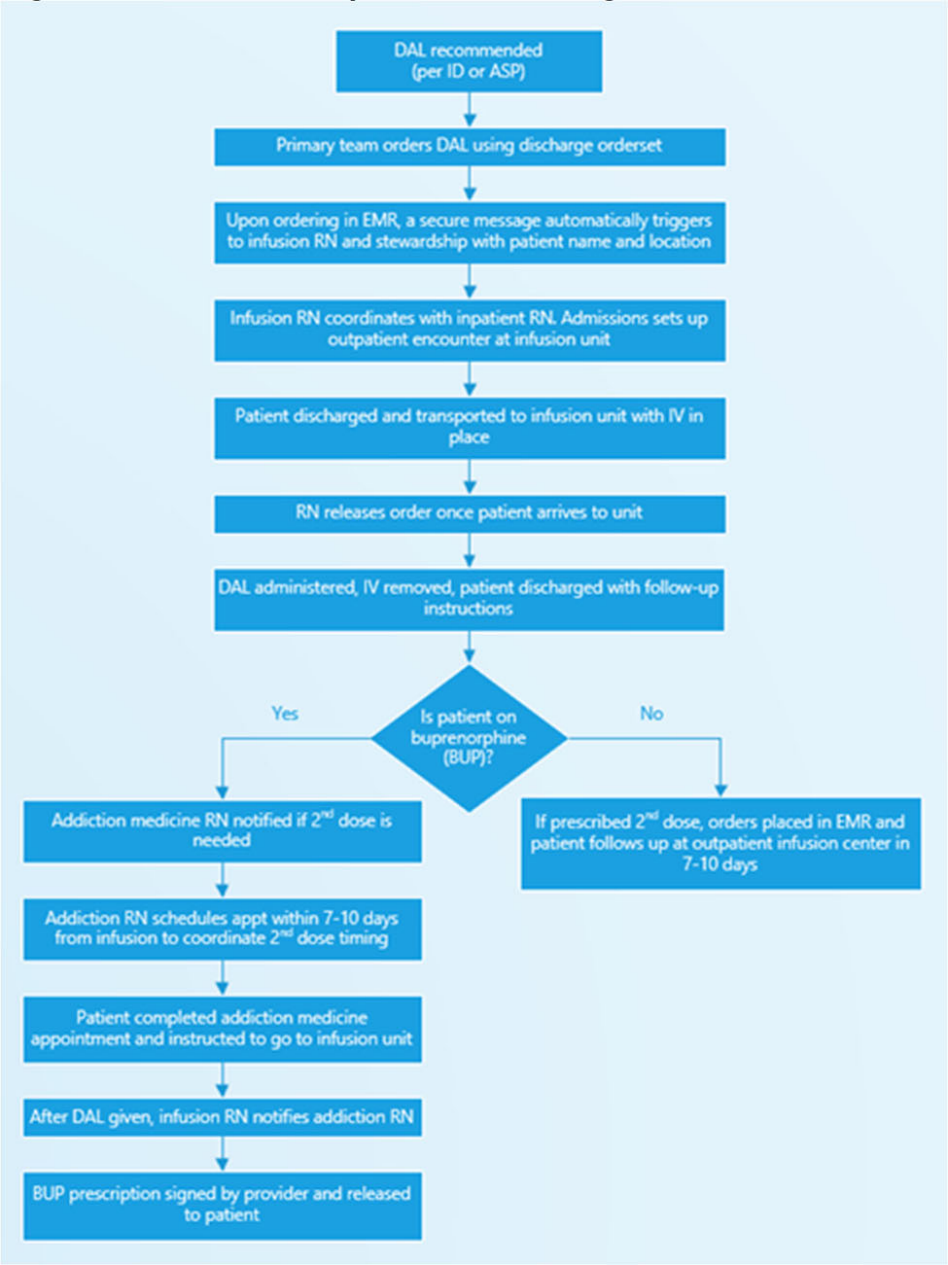
Dalbavancin inpatient to discharge workflow

**Methods:**

A multidisciplinary approach at a tertiary academic healthcare system included nursing, antimicrobial stewardship (ASP), infectious diseases (ID) and addiction medicine (AM) to develop a workflow to identify DAL patients and improve adherence (figure 1). Patients had to be clinically stable with evidence of deep-seated infection from confirmed or strongly suspected Gram-positive organisms with plausible susceptibility to DAL and lack of any reliable oral antibiotic options (feasibility, drug toxicity, drug-drug interactions, resistance, or inability to comply with oral medication due to socioeconomic barriers). DAL had to be recommended by ID or ASP. Patients on buprenorphine (BUP) had their second dose aligned with an AM appointment. Patients were excluded if they left against medical advice prior to DAL, had lack of source control, persistent bacteremia, severe vancomycin allergy, or were discharged to a rehab or skilled nursing facility.

**Table 1:**
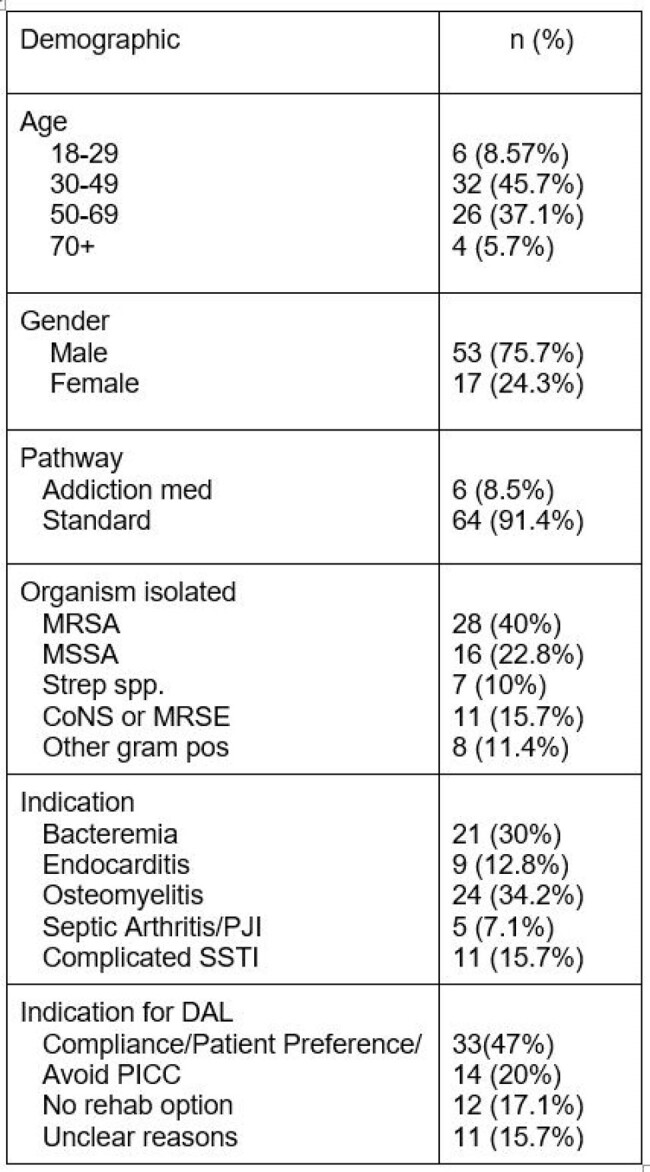
Patient Demographics

**Results:**

Seventy patients were treated with DAL; six patients via the AM pathway and 64 via the standard pathway. In total, 57 patients (81.4%) completed the recommended treatment. In patients who had two doses prescribed, there was 67% completion. In total, 1691 hospital days were avoided. There were 14 readmissions, none were due to DAL failure. Two patients experienced anaphylaxis with their 2^nd^ dose.

**Table 2:** Patient Outcomes

**Figure:**
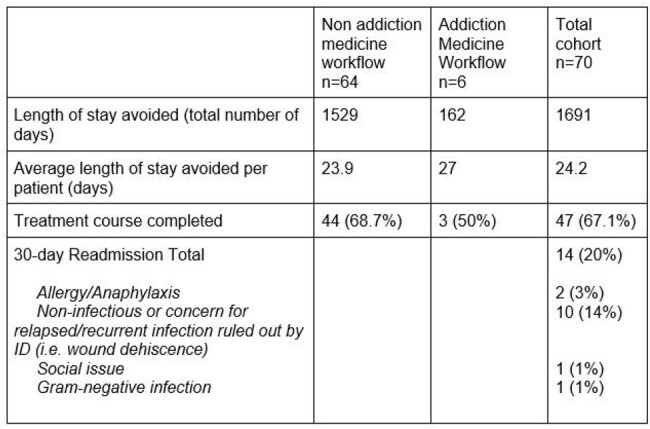

**Conclusion:**

This approach demonstrates that in high-risk populations, DAL can be an effective treatment strategy for invasive infections resulting in high rates of treatment completion. Our data is limited as it does not evaluate outcomes in comparison to standard of care.

**Disclosures:**

Alexandra Hanretty, PharmD, Abbvie: Advisor/Consultant

